# *TOX3* Mutations in Breast Cancer

**DOI:** 10.1371/journal.pone.0074102

**Published:** 2013-09-19

**Authors:** James Owain Jones, Suet-Feung Chin, Li-An Wong-Taylor, Donna Leaford, Bruce A. J. Ponder, Carlos Caldas, Ana-Teresa Maia

**Affiliations:** 1 Cambridge Research Institute, Cancer Research UK, Cambridge, United Kingdom; 2 Department of Oncology, University of Cambridge, Addenbrooke’s Hospital, Cambridge, United Kingdom; 3 Cambridge Experimental Cancer Medicine Centre, Li Ka Shing Centre, Cambridge, United Kingdom; IPATIMUP/Faculty of Medicine of the University of Porto, Portugal

## Abstract

*TOX3* maps to 16q12, a region commonly lost in breast cancers and recently implicated in the risk of developing breast cancer. However, not much is known of the role of *TOX3* itself in breast cancer biology. This is the first study to determine the importance of *TOX3* mutations in breast cancers. We screened *TOX3* for mutations in 133 breast tumours and identified four mutations (three missense, one in-frame deletion of 30 base pairs) in six primary tumours, corresponding to an overall mutation frequency of 4.5%. One potentially deleterious missense mutation in exon 3 (Leu129Phe) was identified in one tumour (genomic DNA and cDNA). Whilst copy number changes of 16q12 are common in breast cancer, our data show that mutations of *TOX3* are present at low frequency in tumours. Our results support that *TOX3* should be further investigated to elucidate its role in breast cancer biology.

## Introduction

We recently performed a genome wide association study using single nucleotide polymorphism (SNP) tagged haplotypes, in which rs3803662 was associated with breast cancer risk [1]. This was the second strongest association identified in this study. rs3803662 tags for a linkage disequilibrium block spanning the 5′ regulatory sequences of the gene *TOX3* (*TOX high mobility group box family member 3*) and the 3′ region of the neighbouring hypothetical gene *LOC643714*. A second genome wide association study [2] identified a significant association between the T allele of rs380662 and the development of oestrogen receptor (ER) positive breast cancer. However, neither *TOX3* nor *LOC643714* has been established as the risk gene inside this interval. More recently, the 16q12 risk locus has been reported to modulate the affinity of FOXA1 binding to chromatin, possibly regulating *TOX3* expression [3].

*TOX3* is located in chromosome 16q12 and consists of seven exons. Although it is predominantly expressed in brain, it is also expressed in breast, with breast tumours expressing it at a higher level than in normal tissue [4], [5]. The protein encoded by *TOX3* contains a high mobility group box (HMG-box) and a glutamine-rich C-terminus (consisting of CAG repeats). It has calcium-dependent transcriptional activity and is a co-factor of CREB and CBP [4], [6], [7].

Loss of heterozygosity (LOH) of the 16q region is commonly observed in breast cancers (33.9% primary tumours), including a 2.3% frequency of homozygous deletions [8]. Several breast cancer cell lines also present chromosomal translocations centromeric to this region [8]. Nevertheless, an important tumour-suppressor gene in this region remains to be identified.

Although *TOX3* falls out of the minimum LOH region, in view of the highly significant association of rs3803662 with breast cancer risk, we hypothesised *TOX3* to be a likely candidate tumour-suppressor gene present on the 16q arm. In the present study we selected 2 sets of primary breast tumours and screened *TOX3* for mutations in the entire coding region, to ascertain whether *TOX3* mutations have a role in breast cancer.

## Materials and Methods

### Ethics Statement

Written informed consent was obtained from all subjects for the collection and research use of breast tumours. Control samples were purified from anonymous waste products of blood donations (leukocyte reduction fliters), and did not require written consent. Both collections were approved by the Addenbrooke’s Hospital Local Research Ethics Committee (REC reference 07/H0308/161 and 04/Q0108/21, breast tumours and blood respectively) and the Nottingham Tenovus Primary Breast Cancer Series.

### Cases and Controls

Patients were recruited from two hospitals: 42 samples were collected from Addenbrookes Hospital and another 96 samples were part of a previously described cohort from Nottingham Hospital [8] (Table S1 – Demographics of sample sets). Lymphocytes of 136 healthy fresh blood donors were used as controls. These samples were collected from anonymous white cell-reduction filters from blood donations were collected from the Blood Centre at Addenbrooke’s Hospital, and lymphocytes were separated by density gradient and magnetic sorting, as previously described [9].

### Nucleic Acid Isolation

DNA from tumours was extracted from 20 sections of 30 µm using the Promega DNA Wizard kit (Promega) according to manufacturer’s instructions. Lymphocyte DNA was extracted by a conventional SDS/proteinase K/phenol method. All DNA samples were quantified with a NanoDrop ND-1000 spectrophotometer (NanoDrop Technologies). Genomic DNA from primary tumours was whole-genome amplified (WGA) using the REPLI-g kit from QIAGEN, according to the manufacturer’s instructions.

Total RNA was extracted from all samples using Qiazol (Invitrogen) following manufacturer’s instructions. The RNA was subsequently treated with DNaseI. cDNA was prepared from 1 µg of total RNA per 20 µl reaction using random hexamers and the Reverse Transcription kit (Applied Biosystems), according to the manufacturer’s instructions, and was diluted in a final volume of 100 µl.

### Mutation Analysis

Whole-genome amplified DNA from primary tumours and genomic DNA from control blood samples were amplified for all seven exons by PCR using primers designed with Primer3 software (sequences provided as File S1). PCR amplification of genomic DNA was carried out for all seven exons in 20 µl reaction containing 10 pmol of each primer, 200 µM of each dNTP (Promega), 1.5 mM MgCl_2_, 1× AmpliTaq Gold buffer II (Applied Biosystems) and 0.1 units of AmpliTaq Gold polymerase (Applied Biosystems). Cycling conditions were 95°C for 5 min, followed by 35–40 cycles consisting of 30 seconds at 95°C, 30 seconds at 56°C and 1 min at 72°C, finishing with a final extension step of 5 min at 72°C. The annealing temperature was 56°C for all exons except for exon1 amplification (58°C), and PCRs for exons 1, 2 and 5 required the addition of the CES additive [10]. cDNA was amplified for all samples with mutations using the KAPA2G Robust PCR kit (KapaBiosystems), following the manufacturer’s instructions. Cycling conditions were 95°C for 3 min, followed by 35 cycles consisting of 15 seconds at 95°C, 15 seconds at 60°C and 15 seconds at 72°C, finishing with a final extension step of 5 min at 72°C. PCR products were purified using either the QIAquick kit (QIAGEN) or using NucleoFast 96-well plates (Macherey-Nagel), and eluted in a final volume of 30 μl.

Sequencing was performed using BigDye Terminator Cycle Sequencing Ready Reaction solution (Applied Biosystems). All products were sequenced in both directions except for exon 7A, which was sequenced only in the forward direction, and therefore repeated twice. All mutations were validated with re-amplification and sequencing of non-WGA tumour DNA and cDNA. The sequencing data was analyzed using DNAStar Lasergene8.0 SeqMan software and compared to the sequences deposited on ENSEMBL (genomic sequence ENSG00000103460, cDNA ENST00000219746 and ENST00000407228). All variants identified in this study were verified in the dbSNP database and the 1000 Genomes Project data.

### Expression Analysis

Expression data for these samples already existed performed on an Agilent platform as reported previously [11].

Relative allelic expression ratios were determined during the sequencing of cDNA samples, by measuring the area under the peaks in the chromatograms for the mutant vs the wild-type bases.

### In-silico Analysis

Candidate deleterious mutations were investigated for putative splice variants, protein structure and function alterations using the Human Splicing Finder, the PSIPRED Protein Structure Prediction Server and SIFT *Sorting Intolerant from Tolerant* web-based software [12]–[14]. All in-silico data is provided as File S2 and S3.

## Results

We screened all seven exons of *TOX3* for mutations in one set of 46 primary tumour samples using Sanger sequencing. The screen of the second set of 96 samples was focused on exons two to seven for which variants were identified in the first set. In total, we acquired good quality data for 133 tumours. Table 1 provides a summary of all identified mutations. The mutation frequency displayed in Table 1 corresponds to the total number of samples for which a successful PCR product was obtained and sequenced for each exon.

**Table 1:** Mutations in *TOX3* in primary breast tumours.

Mutation						Tumour						
Nucleotideand aminoacid	Exon	Codon	Type	EffectPredictiona	Frequency	Typeb	ERStatus	Metastasis	16q12LOH	CDH1Mutation	AllelicExpressionc	#
c.190T>C	3	64	Missense	Positive	1/124	Luminal A	Pos	No	No	No	WT	1
(p.Phe64Leu)												
c.385C>T	3	129	Missense	Positive	1/124	Luminal A	Pos	No	No	No	MUT>WT	2
(p.Leu129Phe)												
						Normal	Pos	No	No	No	WT	3
c.1304C>T^d^	7	435	Missense	Negative	3/133	–	–	No	–	No	MUT = WT	4
(p.Ser435Leu)						Luminal A	Pos	No	Yes	No	MUT	5
c.1525_1554del	7	509–518	Deletion	Positive	1/133	Luminal A	Pos	Yes	Yes	Gln23*	WT	6
(p.Gln509_Gln518del)												

a In-silico prediction (data shown as File S2 and S3).

b PAM50 classification [15], [16].

c WT, only the wild-type allele is detected in the tumour mRNA; MUT, only the mutated allele is detected in the tumour mRNA; MUT = WT and MUT>WT, both mutated and wild-type alleles are detected in the tumour mRNA in equimolar amounts or the mutated allele in higher quantity.

d Reported in COSMIC [20].

**Figure 1:**
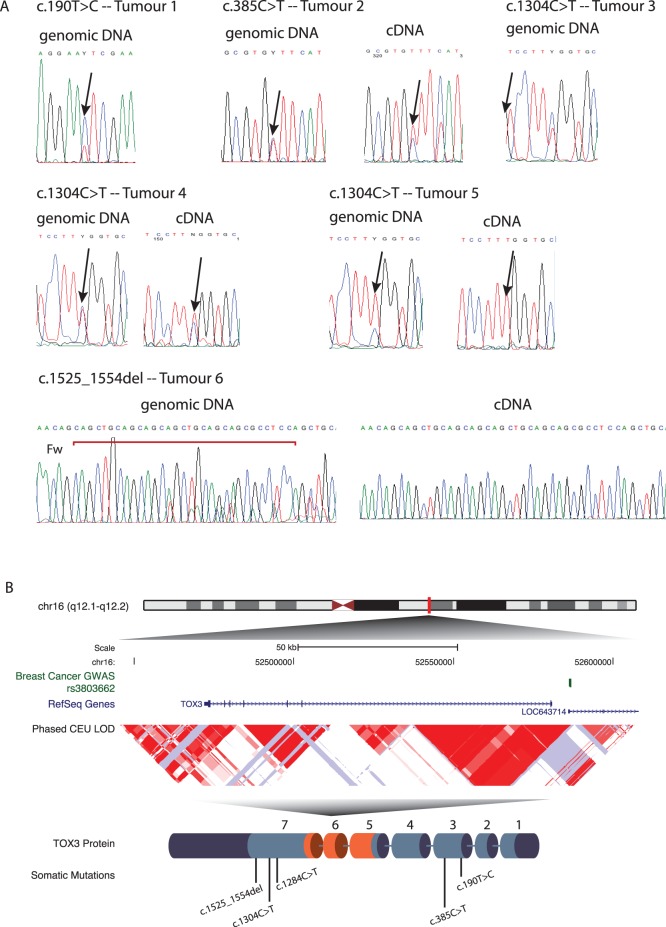
*TOX3* mutations in primary breast tumours. **A)** Direct sequencing profiles of genomic and complementary DNA are shown for all mutations. Arrows indicate the site of the nucleotide change associated with each mutation. **B)** Overview of the *TOX3* mutations identified in breast cancer. Genomic localisation of *TOX3*, relative position to the susceptibility associated SNP rs3803662 (shown in green) and scheme of the protein encoded by *TOX3* with the site of the identified mutations (not to scale). The HMG-box domain is shown in orange.

We found four different mutations in exons three and seven in a total of six tumours: three missense mutations and one deletion (Figure 1).

One tumour sample presented a missense mutation, c.190T>C (Figure 1), leading to a p.Phe64Leu alteration in the protein sequence. This is not predicted to change the folding of TOX3 (PSIPRED) but has a predicted damaging effect according to SIFT. Analysis of the cDNA of the same sample revealed that the mutated allele is not expressed, which suggests that this is a non-deleterious mutation. This tumour is a ER positive Luminal A type tumour.

Another missense mutation was identified in another ER positive Luminal A type tumour, c.385C>T, leading to a leucine to phenylalanine substitution at codon 129 (p.Leu129Phe). This change is not predicted to change the folding of TOX3 according to PSIPRED, but is predicted to have a damaging effect on the protein structure and function according to SIFT. Sequencing of the cDNA of this tumour revealed that the mutated allele is preferentially expressed in the tumour [60% mutant : 40% wild-type], when compared with the allelic proportion of mutant by wild-type in the genomic DNA (Figure1 A). This is potentially a deleterious mutation that warrants further investigation.

A missense mutation in exon seven was detected in three samples, c.1304C>T, resulting in a serine to leucine modification at codon 435 (p.Ser435Leu). According to the in-silico analysis, this mutation is not predicted to modify the folding or function of the TOX3 protein (PSIPRED and SIFT), but interestingly it is differentially expressed between the tumours. A “Normal-like” type ER positive tumour with neutral copy number in this region only expresses the wild-type allele. Another tumour, Luminal A type ER positive, with LOH in 16q12 (tumour 7, Figure 1A) expresses exclusively the mutated allele. This indicates that both alleles of *TOX3* are potentially inactivated in this tumour, one by LOH and the other by point mutation. A third tumour without CGH data available expresses equimolar proportions of mutant and wild-type alleles.

Finally, an in-frame deletion of 30 bp inside exon 7, c.1525_1554del, was detected in one sample with LOH in 16q12 (Figure 1), resulting in the loss of 10 amino acids. This tumour only presented the expression of the wild-type full-length allele, which suggests that this is a non-deleterious mutation. This tumour is o type Luminal A type, ER positive and presented metastasis (not to the bone). This is as well the only tumour for which *CDH1* is mutated (Gln23* nonsense mutation).

The small number of samples with mutations does not permit statistical analysis correlating mutations and clinical characteristics of the tumours. Nevertheless, besides one tumour for which we have very limited information and another which is “normal-like”, all tumours carrying mutations were of the Luminal A type, and are all ER positive [15], [16]. Additionally, two patients presented with metastasis.

To investigate whether the mutational status of these tumours was associated with altered levels of expression of TOX3, we compared mutated vs non-mutated.

## Discussion

To our knowledge this is the first mutation screen of *TOX3* in breast cancer. Our rationale for performing this screen was that *TOX3* maps to a known breast cancer susceptibility locus, which is also commonly a region of LOH in breast cancer. We hypothesised that *TOX3* could be a candidate tumour suppressor gene in 16q.

In our study, we found a frequency of 4.5% coding *TOX3* mutations in primary breast tumours, clustered in exon 3 and in exon 7 (the latter contains the trinucleotide repeat region) (Figure 1B). In-silico predictions indicated that three of the coding mutations have a potential deleterious effect on protein secondary structure or function. Of these, one is expressed by the tumour and therefore potentially pathogenic (p.Leu129Phe, exon 3). The other two are not expressed by the corresponding tumours, and therefore are unlikely to be disease-causing. The variability of preferential expression of the mutant vs wild-type allele in the samples without LOH can be an indication of differential allelic methylation within the tumours, which can lead to loss of expression from one allele. However, all mutations detected in our samples were outside of the HMG-box region (Figure 1B), suggesting that the DNA binding ability of the mutant proteins should not be affected.

The only tumour suppressor gene identified to date in this LOH region in breast tumours is *CDH1* (E-*cadherin*). Mutations in *CDH1* are associated with lobular tumours and have been reported at a frequency of 6.7% [17]. Only one of our samples was also mutated in this gene (Tumour 6, a nonsense mutation). Also, we found that four out of five tumours with *TOX3* mutations, for which we had clinical information, were of Luminal A type. This result suggests that mutations in these two genes might be associated with different sub-types of breast tumours.

The function these mutations might be altering or exerting still remains unclear. Recent reports have provided data that both support and reject the tumour suppressor role of TOX3. Two studies have reported an association of the risk allele of rs3803662 and lower expression of *TOX3* in an allele-specific manner [3], [18]. One of these studies also links the lower expression of *TOX3* with tumour grade and poorer outcome [18]. Interestingly, this study reports a stronger effect of the risk allele of rs3803662 in Luminal A tumours, the same sub-type in which we detect mutations in our study. In our own set, it is unlikely that the mutations are altering the expression of *TOX3*, as we did not find significant expression differences between mutated vs non-mutated samples (Figure S1).

On the other hand, an association between *TOX3* overexpression in tumours and lower *BRCA1* expression and tumour aggressiveness has been reported recently [19]. Nevertheless, this study also reports genomic amplification of *TOX3* in advanced breast tumours, which we did not detect in our own samples. It is possible that *TOX3* might play a complex role in promoting tumour development or protecting against it in a subtype-specific manner.

It has also been shown that the effects of *TOX3* expression and of the risk allele of rs3803662 in breast cancer is stronger in ER positive tumours [1], [4], [18]. Interestingly, all the samples in which we detected mutations are also ER-positive. Thus, our data further supports the link between *TOX3* and oestrogen-dependent transcription.

In conclusion, our study reveals that *TOX3* is mutated in breast tumours, albeit at a low frequency. Of the four mutations identified in this study, three are expressed in the tumours and one is potentially deleterious. These results add to the evidence that *TOX3* is associated with breast cancer, but require validation in a larger set to clarify the role of these mutations in tumour development and progression.

## Supporting Information

Figure S1 Expression of *TOX3* in sample with and without mutations.
(EPS)

Table S1 Demographics of samples sets.
(DOC)

File S1 Oligonucleotide sequences.
(TIFF)

File S2 SIFT Prediction Results, for the missense mutations based on the sequences of the two TOX3 variants (ENSP00000385705, ENSP00000219746).
(TIFF)

File S3 PSIPRED Prediction Results, for the missense and deletion mutations.
(TIFF)

## References

[1] Easton DF, Pooley KA, Dunning AM, Pharoah PDP, Thompson D, et al (2007) Genome-wide association study identifies novel breast cancer susceptibility loci. Nature 447: 1087–1093. 17529967 10.1038/nature05887 PMC2714974

[2] Stacey SN, Manolescu A, Sulem P, Rafnar T, Gudmundsson J, et al (2007) Common variants on chromosomes 2q35 and 16q12 confer susceptibility to estrogen receptor-positive breast cancer. Nat Genet 39: 865–869. 17529974 10.1038/ng2064

[3] Cowper-Sal Lari R, Zhang X, Wright JB, Bailey SD, Cole MD, et al (2012) Breast cancer risk-associated SNPs modulate the affinity of chromatin for FOXA1 and alter gene expression. Nat Genet 44: 1191–1198. 23001124 10.1038/ng.2416 PMC3483423

[4] Dittmer S, Kovacs Z, Yuan SH, Siszler G, Kögl M, et al (2011) TOX3 is a neuronal survival factor that induces transcription depending on the presence of CITED1 or phosphorylated CREB in the transcriptionally active complex. J Cell Sci 124: 252–260. 21172805 10.1242/jcs.068759

[5] Udler MS, Ahmed S, Healey CS, Meyer K, Struewing J, et al.. (2010) Fine Scale mapping of the breast cancer 16q12 locus. Hum Mol Genet. 10.1093/hmg/ddq122 PMC2876886 20332101

[6] O’Flaherty E, Kaye J (2003) TOX defines a conserved subfamily of HMG-box proteins. BMC Genomics 4: 13. 12697058 10.1186/1471-2164-4-13 PMC155677

[7] Yuan SH, Qiu Z, Ghosh A (2009) TOX3 regulates calcium-dependent transcription in neurons. Proc Natl Acad Sci USA 106: 2909–2914. 19196971 10.1073/pnas.0805555106 PMC2650364

[8] Chin SF, Teschendorff AE, Marioni JC, Wang Y, Barbosa-Morais NL, et al (2007) High-resolution aCGH and expression profiling identifies a novel genomic subtype of ER negative breast cancer. Genome Biol 8: R215. 17925008 10.1186/gb-2007-8-10-r215 PMC2246289

[9] Maia AT, Spiteri I, Lee AJ, O’Reilly M, Jones L, et al (2009) Extent of differential allelic expression of candidate breast cancer genes is similar in blood and breast. Breast Cancer Res 11: R88. 20003265 10.1186/bcr2458 PMC2815552

[10] Ralser M, Querfurth R, Warnatz HJ, Lehrach H, Yaspo ML, et al (2006) An efficient and economic enhancer mix for PCR. Biochem Biophys Res Commun 347: 747–751. 16842759 10.1016/j.bbrc.2006.06.151

[11] Naderi A, Teschendorff AE, Barbosa-Morais NL, Pinder SE, Green AR, et al (2007) A gene-expression signature to predict survival in breast cancer across independent data sets. Oncogene 26: 1507–1516. 16936776 10.1038/sj.onc.1209920

[12] Desmet FO, Hamroun D, Lalande M, Collod-Béroud G, Claustres M, et al (2009) Human Splicing Finder: an online bioinformatics tool to predict splicing signals. Nucleic Acids Res 37: e67. 19339519 10.1093/nar/gkp215 PMC2685110

[13] Bryson K, McGuffin LJ, Marsden RL, Ward JJ, Sodhi JS, et al (2005) Protein structure prediction servers at University College London. Nucleic Acids Res 33: W36–38. 15980489 10.1093/nar/gki410 PMC1160171

[14] Ng PC, Henikoff S (2003) SIFT: Predicting amino acid changes that affect protein function. Nucleic Acids Res 31: 3812–3814. 12824425 10.1093/nar/gkg509 PMC168916

[15] Perou C, Sorlie T, Eisen M, van de Rijn M, Jeffrey S, et al (2000) Molecular portraits of human breast tumours. Nature 406: 747–752. 10963602 10.1038/35021093

[16] Sorlie T, Perou C, Tibshirani R, Aas T, Geisler S, et al (2001) Gene expression patterns of breast carcinomas distinguish tumor subclasses with clinical implications. Proc Natl Acad Sci U S A 98: 10869–10874. 11553815 10.1073/pnas.191367098 PMC58566

[17] Boyault S, Drouet Y, Navarro C, Bachelot T, Lasset C, et al (2012) Mutational characterization of individual breast tumors: TP53 and PI3K pathway genes are frequently and distinctively mutated in different subtypes. Breast Cancer Res Treat 132: 29–39. 21512767 10.1007/s10549-011-1518-y

[18] Gudmundsdottir ET, Barkardottir RB, Arason A, Gunnarsson H, Amundadottir LT, et al (2012) The risk allele of SNP rs3803662 and the mRNA level of its closest genes TOX3 and LOC643714 predict adverse outcome for breast cancer patients. BMC Cancer 12: 621. 23270421 10.1186/1471-2407-12-621 PMC3553017

[19] Shan J, Dsouza SP, Bakhru S, Al-Azwani EK, Ascierto ML, et al (2013) TNRC9 downregulates BRCA1 expression and promotes breast cancer aggressiveness. Cancer Res 73: 2840–2849. 23447579 10.1158/0008-5472.CAN-12-4313

[20] Forbes SA, Bindal N, Bamford S, Cole C, Kok CY, et al (2011) COSMIC: mining complete cancer genomes in the Catalogue of Somatic Mutations in Cancer. Nucleic Acids Res 39: D945–950. 20952405 10.1093/nar/gkq929 PMC3013785

