# Supplementary material for: TOX3 Mutations in Breast Cancer
Source: PLoS One. 2013 Sep 19;8(9):e74102. doi: 10.1371/journal.pone.0074102 (PMC3777980; doi:10.1371/journal.pone.0074102)
Supplement: Table S1 — Demographics of samples sets. (DOC) [file pone.0074102.s002.doc]

**Table S1: Demographics of patients used in this study**

**Addenbrookes Hospital Set**

| **Parameter** | **Numbers** |
| --- | --- |
| No of Patients | 42 |
| Median age, years (range) | 57 (37-87) |
| Tumor size, cm (range) | 2.2 (1-5.2) |
| Patient Status (*%*) |  |
| Alive | 29 (*69*) |
| Dead | 9 (*21*) |
| No Information | 4 (*10*) |
| Menopause Status (*%*) |  |
| Pre | 18 (43) |
| Post | 10 (*22*) |
| No Information | 4 (*10*) |
| Pathologic Grade (*%*) |  |
| I | 4 (*10*) |
| II | 10 (*24*) |
| III | 20 (*48*) |
| No Information | 4 (*10*) |
| Lymph Node Status (*%*) |  |
| Negative | 14 (*33*) |
| Positive | 23 (*55*) |
| No Information | 5 (*12*) |
| Vascular Invasion (*%*) |  |
| No | 15 (*69*) |
| Yes | 17 (*31*) |
| Unsure | 2 (*5*) |
| No Information | 8 (*20*) |
| ER Status (*%*) |  |
| Positive | 32 (*76*) |
| Negative | 5 (*12*) |
| No Information | 5 (1*2*) |
| Nottingham Prognostic Index (%) |  |
| ≤3.4 | 2 (*5*) |
| >3.4 | 11 (*26*) |
| No Information | 29 (69) |

**Nottingham Hospital Set**

| **Parameter** | **Numbers** |
| --- | --- |
| No of Patients | 148 |
| Median age, years (range) | 58 (35-70) |
| Survival/months, median (range) | 134 (7-161) |
| Tumor size, cm (range) | 1.8 (0.1-4.5) |
| Patient Status (*%*) |  |
| Alive | 93 (*63*) |
| Dead from Breast Cancer | 37 (*25*) |
| Dead from Other Causes | 15 (*10*) |
| Lost to follow up | 3 (*2*) |
| Menopause Status (*%*) |  |
| Pre | 48 (*32*) |
| Post | 100 (*68*) |
| Pathologic Grade (*%*) |  |
| I | 34 (*23*) |
| II | 57 (*39*) |
| III | 57 (*39*) |
| Lymph Node Status (*%*) |  |
| Negative | 103 (*70*) |
| Positive | 45 (*30*) |
| Recurrence (*%*) |  |
| No | 100 (*68*) |
| Yes | 48 (*32*) |
| Distant Metastases (*%*) |  |
| No | 114 (*77*) |
| Yes | 34 (*23*) |
| Vascular Invasion (*%*) |  |
| No | 102 (*69*) |
| Yes | 46 (*31*) |
| ER Status (*%*) |  |
| Positive | 100 (*68*) |
| Negative | 45 (*30*) |
| No Information | 3 (*2*) |
| Nottingham Prognostic Index (%) |  |
| ≤3.4 | 63 (*43*) |
| >3.4 | 85 (*57*) |
