# Supplementary figures and images for: TOX3 Mutations in Breast Cancer
Source: PLoS One. 2013 Sep 19;8(9):e74102. doi: 10.1371/journal.pone.0074102 (PMC3777980; doi:10.1371/journal.pone.0074102)

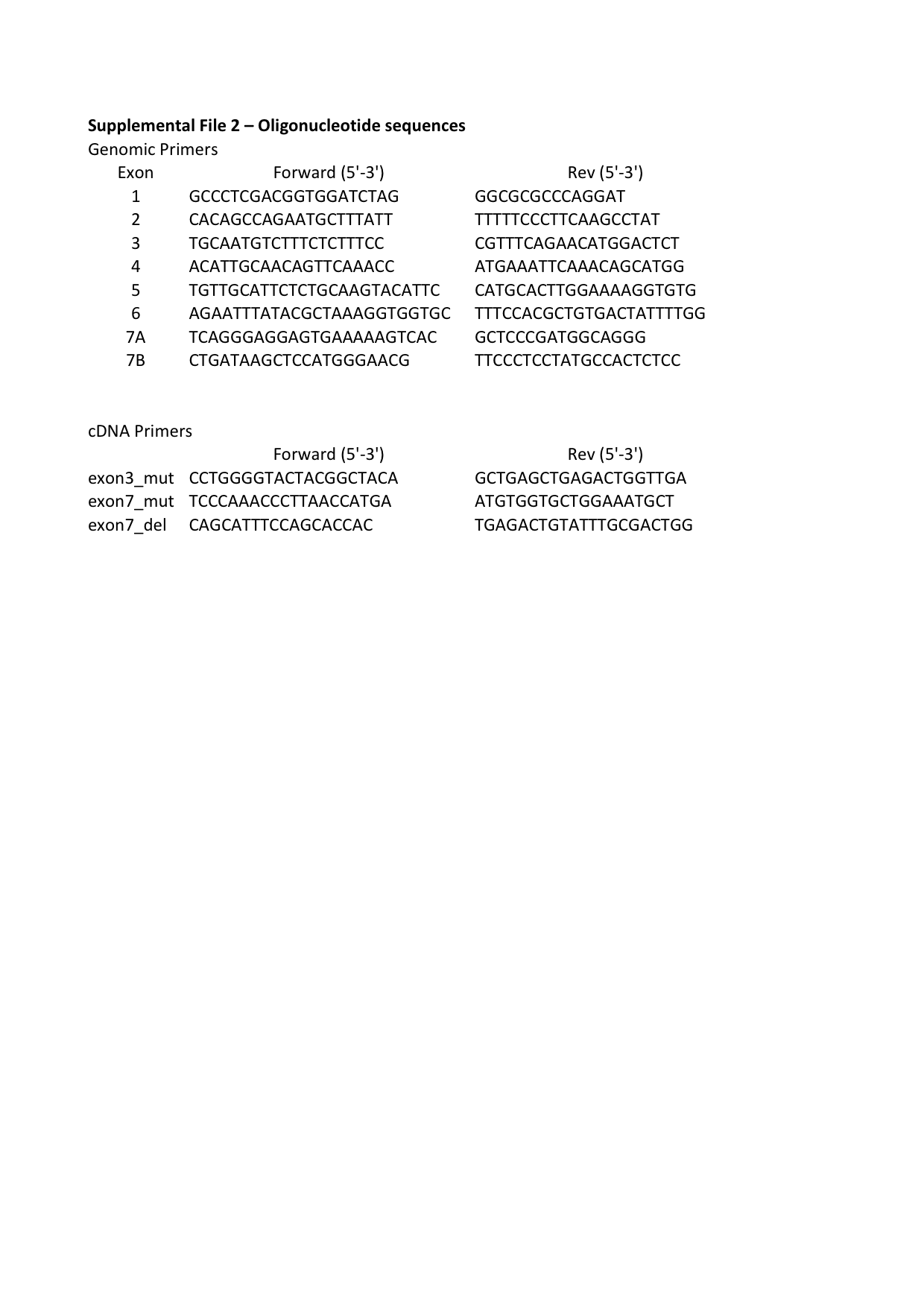

Supplement: File S1 — Oligonucleotide sequences. (TIFF) [file pone.0074102.s003.tiff]

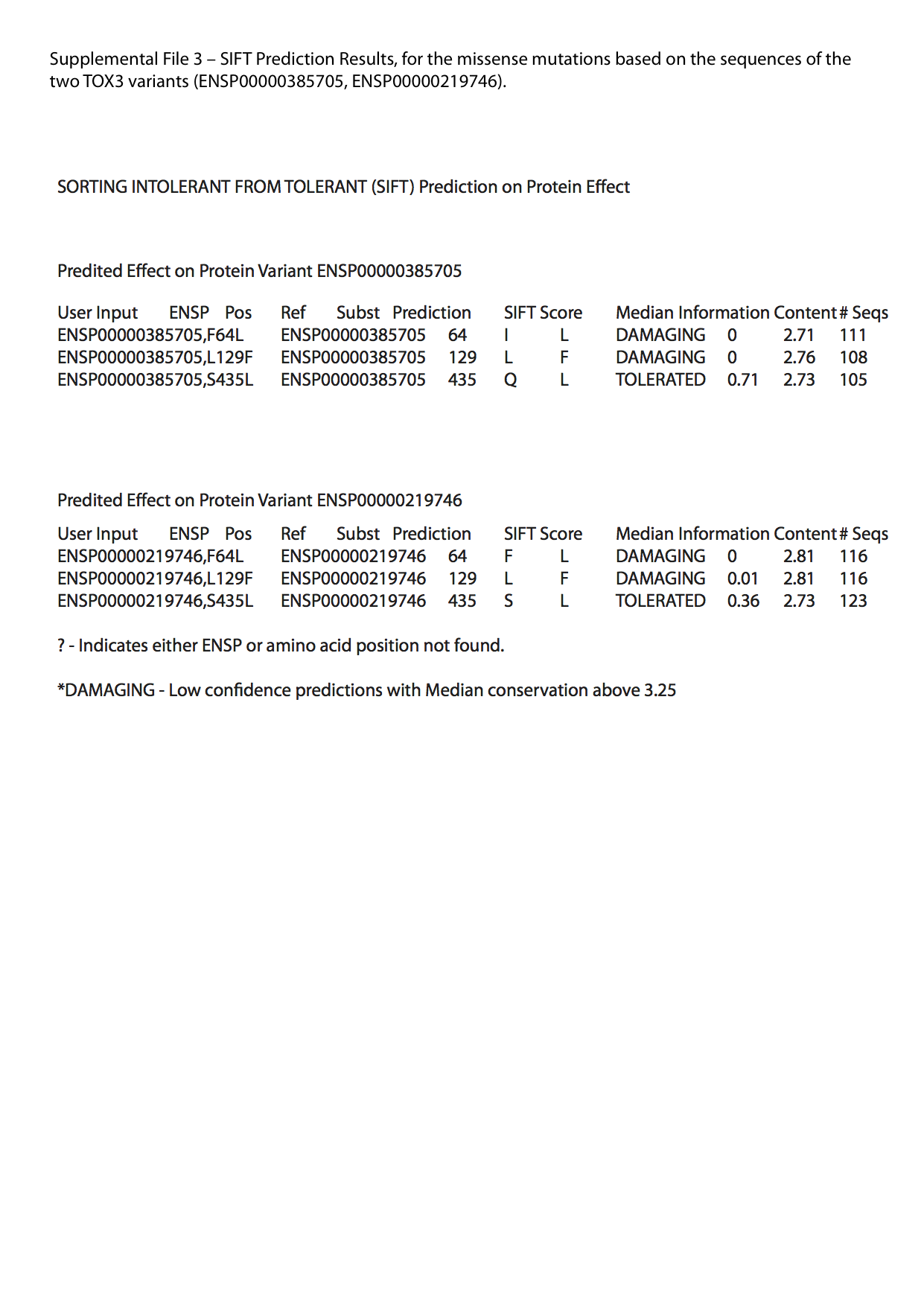

Supplement: File S2 — SIFT Prediction Results, for the missense mutations based on the sequences of the two TOX3 variants (ENSP00000385705, ENSP00000219746). (TIFF) [file pone.0074102.s004.tiff]

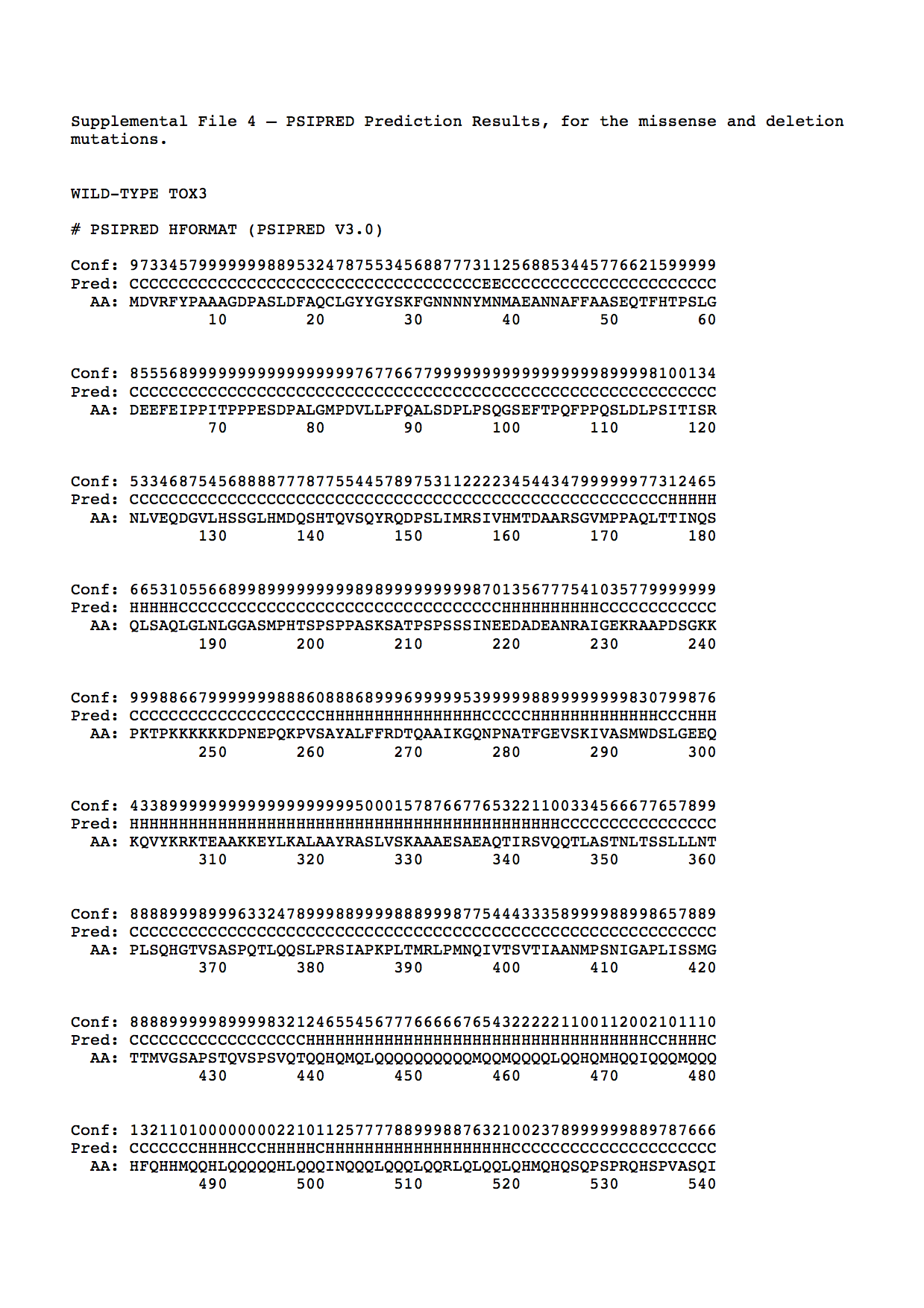

Supplement: File S3 — PSIPRED Prediction Results, for the missense and deletion mutations. (TIFF) [file pone.0074102.s005.tiff]
